# Phylogeography of *Dendrolimus punctatus* (Lepidoptera: Lasiocampidae): Population differentiation and last glacial maximum survival

**DOI:** 10.1002/ece3.5278

**Published:** 2019-06-19

**Authors:** Jing Li, Qian Jin, Geng‐ping Zhu, Chong Jiang, Ai‐bing Zhang

**Affiliations:** ^1^ College of Life Sciences Capital Normal University Beijing China; ^2^ Suqian Institute of Agricultural Sciences Jiangsu Academy of Agricultural Sciences Suqian China; ^3^ College of Life Sciences Tianjin Normal University Tianjin China

**Keywords:** *Dendrolimus punctatus*, DNA barcode, phylogeography, population expansion, quaternary climate oscillation

## Abstract

Although the Masson pine moth, *Dendrolimus punctatus*, is one of the most destructive forest pest insects and is an endemic condition in China, we still do not fully understand the patterns of how its distribution range varies in response to Quaternary climatic oscillations. Here, we sequenced one maternally inherited mitochondrial gene (*COI*) and biparentally inherited nuclear data (*ITS1* and *ITS2*) among 23 natural populations across the entire range of the species in China. A total of 51 mitotypes and 38 ribotypes were separately obtained using mtDNA and *ITS1* data. Furthermore, significant phylogeographical structure (*N*_ST_ > *G*_ST_, *p* < 0.01) were detected. The spatial distribution of mitotypes implied that two distinct groups existed in the species: one in the southwest distribution, including 10 locations, and the other located in the northeast region of China. It is suggested, therefore, that each group was derived from ancestors that occupied different isolated refugia during previous periods, possibly last glacial maximum. Mismatch distribution and Bayesian population dynamics analysis suggested the population size underwent sudden expansion, which is consistent with the results of ecological niche modeling. As a typical phytophagous insect, the history of population expansion was in accordance with the host plants, providing abundant food resources and habitat. Intraspecific success rate of barcoding identification was lower than interspecific ones, indicating a level of difficulty in barcoding individuals from different populations. However, it still provides an early insight into the pattern of genetic diversity within a species.

**OPEN RESEARCH BADGES:**

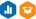

This article has been awarded an Open Data and Open Materials. All materials and data are publicly accessible via the Open Science Framework at https://doi.org/10.5061/dryad.2df87g2. Learn more about the Open Practices badges from the Center for Open Science: https://osf.io/tvyxz/wiki.

## 1 INTRODUCTION

It has been widely accepted that the distribution and genetic structure of current organism have been greatly affected by environmental factors and their historical processes (Hewitt, 2000). The oscillations of Quaternary climate changes played critical roles in multiple contraction–expansion processes, which have greatly shaped current geographical distributions and genetic differentiations of many species in temperate zones of East Asian (Hickerson et al., 2010; Qiu, Fu, & Comes, 2011). However, compared with the numerous studies focused in the Qinghai‐Tibet Plateau and Mts. Hengduan (Lei, Qu, Song, Alström, & Fjeldså, 2015; Liu et al., 2016; Wang et al., 2018), such investigations have just started in the subtropical regions of China. During the Last Glacial Maximum (LGM) period, the climate of this region became much cooler and dryer, which drove the southward migration of the warm‐temperate evergreen forests as far as c.24°N (Harrison, Yu, Takahara, & Prentice, 2001; Qiu et al., 2011). Moreover, the vast continental hilly areas in the subtropical China provided potential refugia for temperate deciduous forests and boreal conifer trees (Jiang et al., 2016; Lei et al., 2015).

**Figure 1:**
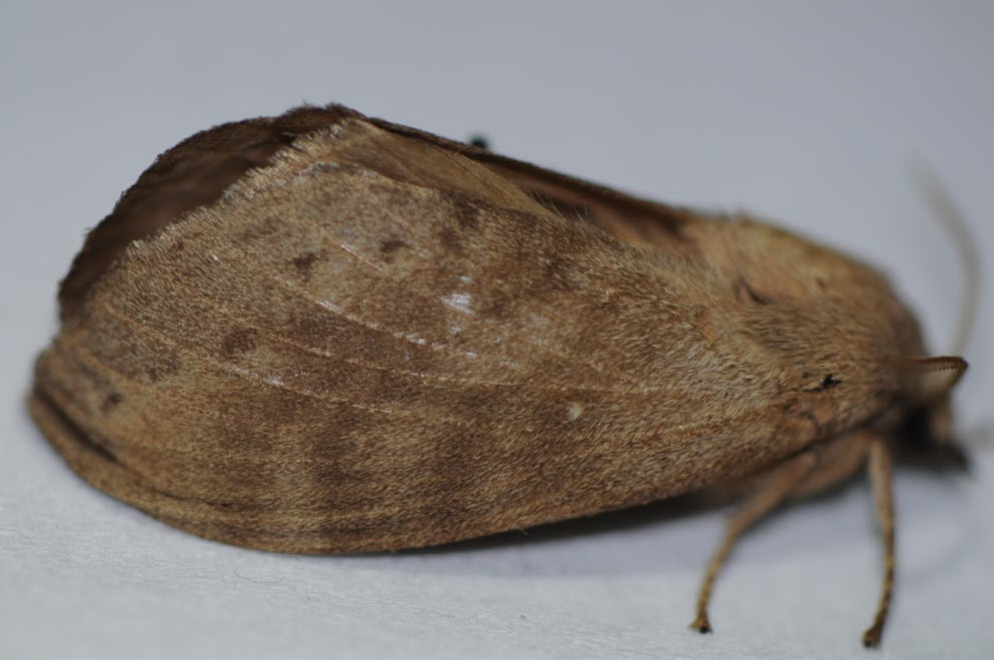
Photograph of *Dendrolimus punctatus* (Walker, 1985) (by Dr. Chong‐Hui Yang)

Previous research implied that much of the genetic variety of phytophagous insects was motivated by the diverging specialization processes onto different host plants (Peterson & Denno, 1998). More detailed studies based on population‐level variations of host plants implied that the diversification of host could have an influence on the differentiation of insects (Friberg, Schwind, & Roark, 2014; Friberg, Schwind, & Thompson, 2016; Matsubayashi, Ohshima, & Nosil, 2010; Singer, Ng, & Moore, 1991). *Dendrolimus punctatus* (Walker, 1985) distributed in subtropical China was adopted in our study to identify whether the genetic differentiation of host plants, caused by climate changes during the ice age, affects genetic variation of their predators, the phytophagous insects. *Dendrolimus* species (Lepidoptera: Lasiocampidae) are one of the most serious phytophagous pests worldwide and caused extensive forest damage (Dai et al., 2012; Zhao, Wu, Lv, Chen, & Lin, 1999; Zhang, Wang, Tan, & Li, 2003; Zhang, Li, Chen, & Zhang, 2004) (Figure 1). *Dendrolimus punctatus* is endemic to southern Asia and, in recent years, has become the most serious and economically damaging insect pest in the south China forests. *Pinus massoniana*, which is a typical warm‐temperate evergreen coniferous in south mainland China, is the main host plant of *D. punctatus* (Cai, 1995; Hou, 1987). Based on a phylogeographic analysis, *P. massoniana* experienced a recent expansion of its smaller populations during the Quaternary period (Ge et al., 2012). The increasing population density, which increased rapidly after a few generations, posed a great threat to forestry production. Consequently, great efforts were devoted to keep the size of population at a relatively low and stable level. Notably, low‐density populations are difficult to detect in the field, which makes it extremely arduous to collect samples.

Here, we examined the genetic variation across the distribution areas of *D. punctatus* with the barcode fragment of the *COI* gene as well as two nuclear loci: ribosomal first internal transcribed spacer (rDNA *ITS1*) and second spacer (rDNA *ITS2*). Two different inherited molecular markers were included in this study: mtDNA marker (*COI*) represented maternal inheritance; the *ITS* fragments represented the biparental inheritance. In addition, these three molecular markers shared barcoding sequences well and proved to be excellent methods to help explain the interspecific relationship among the sibling species of *D. punctatus* (Dai et al., 2012). Nevertheless, although DNA barcoding has been widely used as a concept to facilitate biological identifications at the species level (Hebert, Cywinska, Ball, & deWaard, 2003), there has been limited use related to lower categories such as subspecies and populations (Huemer & Hebert, 2011; Valade et al., 2009). These lower categories, defined by evidence of geographical, morphological, and ecological criteria, can be potential or cryptic species (DeSalle, 2006). It would be particularly desirable to test whether these widely accepted interspecific barcodes could also be adopted as population identification. Although the typical DNA barcoding was not sufficient to identify geographical clusters within species (Hajibabaei, Singer, Hebert, & Hickey, 2007), the limited geographical isolation was observed between different regions (Rach, DeSalle, Sarkar, Schierwater, & Hadrys, 2008; Zhao et al., 1999). The success rate of barcoding at lower categories largely depends on the phylogeographic structure of the taxon (Zhang et al., 2003). Factors such as the geographic distribution of the populations, time and degree of isolation, effective population sizes, and particularly gene flow showed fundamental effects. Therefore, our present work seeks to address the following points:

1. to investigate the phylogeographical structure of the masson pine pest;
2. to test whether the demographic history of this moth corresponds to the “contraction‐expansion” mode similar to its host plant;
3. to explore the potential availability of population identification using the DNA barcode.

## 2 MATERIALS AND METHODS

### 2.1 Specimen sampling

**Figure 2:**
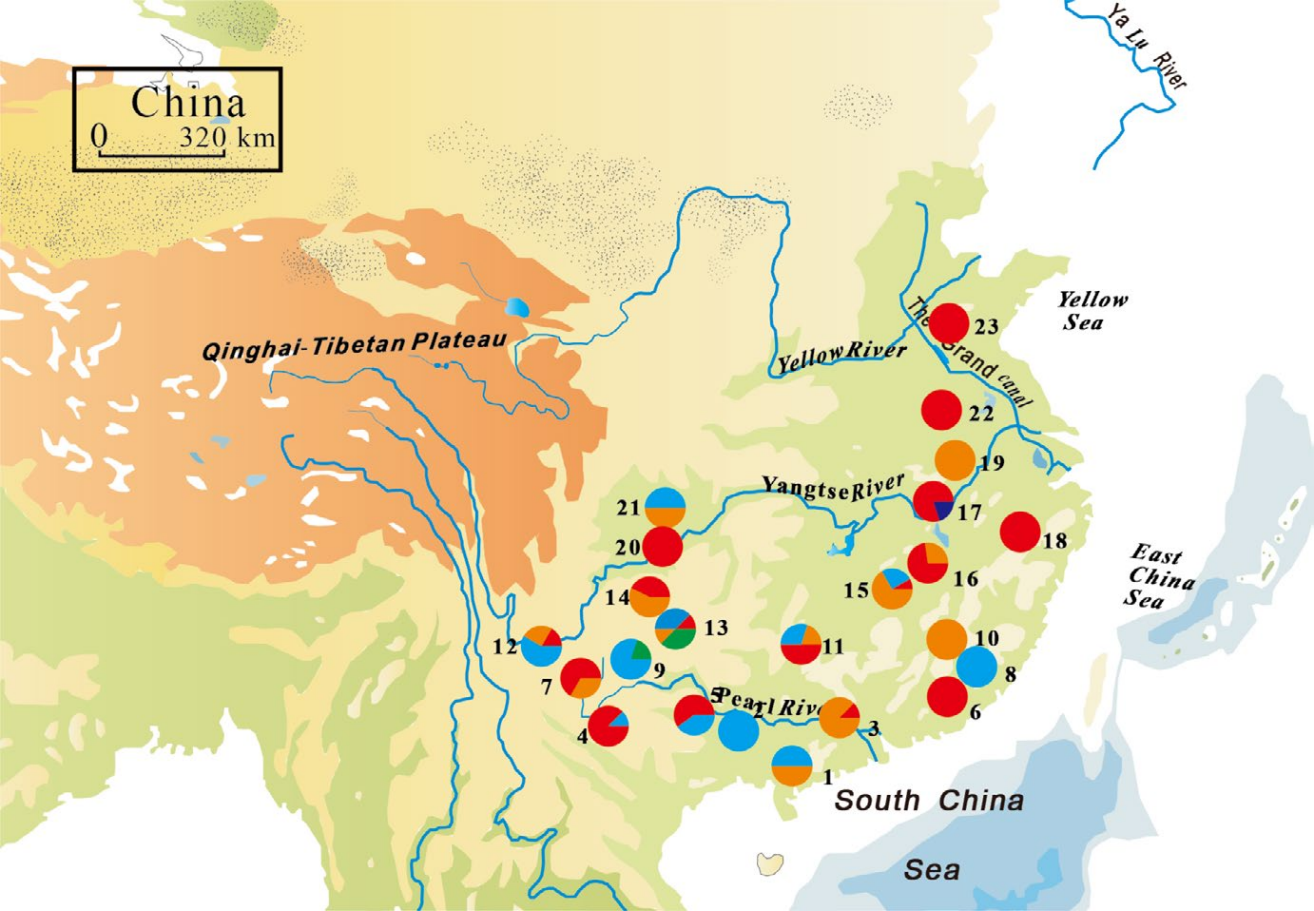
A map of the sampling sites and the geographic distribution of *Dendrolimus punctatus* mtDNA haplotypes. Pie charts show the proportion of mitotypes within each population, and colors represent different lineages. The numbers beside the circles represent population numbers listed in Table 1

The specimens of *D. punctatus* were collected as larvae, which were gathered with tweezers during the day. The larvae of *D. punctatus* displayed an aggregated distribution pattern. Hence, one larva of pine caterpillar was collected every 5 m. The sampling scheme, including a total of 236 specimens from 23 localities, covered the major geographic distribution of this species (Figure 2; Table 1). The specimens were collected in the natural forests. In order to preserve DNA sequence integrity, the larvae collected in natural forests were immediately placed in 95% ethanol and ultimately stored at 4°C. Voucher specimens were deposited at the Capital Normal University, Beijing, China.

**Table 1:** Locations of populations of *Dendrolimus punctatus* sampled, sample sizes (N), frequencies of mtDNA haplotypes per population, and estimates of haplotype diversity (*h*), and nucleotide diversity (*π*) for mitotypes

Pop.	Abbr.	Locality	Lat. (N)	Long. (E)	*N*	Nh	Haplotypes nos.	*S*	*h* (*SD*)	*π* in ‰
1	GXLC	Luchuan, Guangxi	22.19	110.16	6	4	H6 (2). H11 (2). H13(1). H18 (1)	2	0.867(0.129)	1.93
2	GXNN	Naning, Guangxi	22.49	108.21	12	4	H14(8). H15 (2). H16 (1). H17 (1)	3	0.561(0.154)	1.62
3	GDDQ	Deqing, Guangdong	23.15	111.77	8	5	H 11(3). H22(2). H23 (1). H24 (1). H25 (1)	3	0.857(0.108)	2.37
4	YNWS	Wenshan, Yunnan	23.22	104.15	6	4	H1 (1). H2 (3). H3 (1). H4 (1)	5	0.8(0.172)	3.51
5	GXBS	Baise, Guangxi	23.53	106.37	10	3	H46 (1). H47 (5). H48(4)	8	0.644(0.101)	6.2
6	GDXN	Xingning, Guangdong	24.15	115.73	4	2	H2(2). H7(2)	1	0.667(0.204)	1.13
7	YNSL	Shilin, Yunnan	24.45	103.16	6	2	H2 (4). H41 (2)	3	0.533(0.172)	3.4
8	FJSH	Shanghang, Fujian	25.02	116.25	9	3	H38 (5). H39 (1). H40 (3)	2	0.639(0.126)	1.23
9	GZXY	Xingyi, Guizhou	25.05	104.54	5	3	H 6(1). H19 (3). H20 (1)	12	0.7(0.218)	8.16
10	FJWP	Wuping, Fujian	25.10	116.10	11	6	H11 (3). H18 (2). H22 (3). H35 (1). H36 (1). H37 (1)	4	0.873(0.071)	2.7
11	GXQZ	Quanzhou, Guangxi	25.55	111.04	20	8	H5 (1). H6 (2). H8 (8). H9 (2). H10 (1). H11 (4). H12 (1). H13 (1)	7	0.811(0.071)	4.87
12	YNYR	Yongren, Yunnan	26.03	101.40	12	4	H4 (3). H5 (1). H6 (7). H7 (1)	3	0.636(0.128)	1.49
13	GZGY	Guiyang, Guizhou	26.38	106.37	8	4	H5 (1). H6 (3). H18 (1). H19 (3)	11	0.786(0.113)	102
14	GZHZ	Hezhang, Guizhou	27.07	104.43	7	3	H6 (3). H11 (3). H21 (1)	3	0.714(0.127)	1.94
15	JXYC	Yichun, Jiangxi	27.47	114.23	12	5	H5 (1). H6 (1). H22 (4). H23 (4). H45 (2)	5	0.803(0.078)	3.27
16	JXGA	Gaoan, Jiangxi	28.25	115.22	11	6	H2(4). H3(1). H41(2). H42(2). H43(1). H44(1)	5	0.855(0.085)	3.83
17	ZJJS	Jiangshan, Zhejiang	28.44	118.37	10	4	H27(3). H28(2). H29(4). H30(1).	6	0.778(0.091)	4.72
18	ZJQX	Quxian, Zhejiang	29.03	119.11	11	4	H1(1). H2(6). H3(3). H26(1)	3	0.673(0.123)	1.48
19	ZJLX	Lanxi, Zhejiang	29.12	119.28	9	6	H11(2). H18(1). H31(3). H32(1). H33(1). H34(1)	8	0.889(0.091)	6.8
20	SCLX	Luxian, Sichuan	29.15	105.38	4	1	H2(4)	0	0	0
21	SCHY	Huaying, Sichuan	30.38	106.77	8	5	H6(3). H9(1). H22(2). H23(1). H37(1)	5	0.857(0.108)	3.46
22	AHQS	Qianshan, Anhui	30.57	116.34	12	3	H2(10). H3(1). H46(1)	2	0.318(0.164)	0.57
23	HBCD	Chengde, Hebei	40.59	118.53	35	3	H49(15). H50(8). H51(12)	2	0.666(0.032)	1.65

Note

Voucher specimens are deposited in the Herbarium of Capital Normal University (CNU).

### 2.2 DNA extraction, PCR amplification, and sequencing

DNA samples were isolated from the legs of adult individuals using DNeasy Blood and Tissue kit (Biomed) following the manufacturer's protocol. Sequences of mitochondrial gene *COI* and nuclear *ITS* were amplified using rTaq (Takara). The same PCR primers and amplification reaction of Dai et al. (2012) were used in our study. The amplification products were subjected to electrophoresis in a 2% (w/v) agarose gel in TAE buffer (0.04 M *Tris*–acetate, 0.001 M EDTA) to check whether the amplification reactions were successful. Then, the amplification products were used for sequencing, which was performed with an ABI3130 sequencer at Biomed Biotechnology Co., Ltd. All sequences have been deposited in GenBank (accession numbers: KF366914–KF367149 for *COI*, KF367150–KF367271 for *ITS1*, and KF367272–KF367452 for *ITS2*), and the *COI* barcodes were also submitted to the BOLD system (accession numbers: GBGL14669–GBGL14904).

### 2.3 Phylogenetic analysis and network reconstruction

The raw DNA sequences were all checked manually and aligned with software MUSCLE (Edgar, 2004). Aligned *COI* sequences were translated into amino acid sequences using the MEGA program (Kumar, Tamura, & Nei, 2004). The phylogenetic relationships within *D. punctatus* were reconstructed using maximum likelihood (ML) inference and Bayesian inference (BI). Two specimens from species *Dendrolimus superans* (Butler, 1877) were used as outgroups. The best‐fit model of nucleotide substitution was selected from 88 models using the Akaike information criterion (AIC) with jModelTest 2 (Darriba, Taboada, Doallo, & Posada, 2012). The ML analysis was conducted using the program RAxML v.7.0.4 with 1,000 bootstraps conducted (Alexandros, 2006). The GTR+G model was used for all genes. The Bayesian analysis was performed using MrBayes v3.1.2 (Ronquist & Huelsenbeck, 2003). The MCMC analysis was run for 10,000,000 generations, following a burn‐in series of 1,000 generations. Haplotype networks for *COI* sequence and each *ITS* dataset were constructed to better visualize the reticular relationships of *D. punctatus*. The median‐joining (MJ) haplotype networks were constructed to draw an unrooted network with Network 5 (Fluxus Technology Ltd.) based on *COI* gene, as well as *ITS1* and *ITS2*.

### 2.4 Population genetic analysis

All analyses were executed separately for the *COI* gene and the *ITS* fragments. We used DnaSP 5.10 (Librado & Rozas, 2009) to assess population genetic diversity, including the number of sequences (*N*), number of haplotypes (*N*h), haplotype diversity (*h*), and nucleotide diversity (*π*). The program PERMUT (available at www.pierroton.inra.fr/genetics/labo/Software/) was applied to estimate the average gene diversity within a population (*H*_S_), total gene diversity (*H*_T_), and between‐population divergence (*G*_ST_, *N*_ST_) with 1,000 permutations tests.

Analysis of molecular variance (AMOVA), the average values of population differentiation (*F*_ST_), and Mantel test were all calculated to detect the population structure with Arlequin V3.5 software (Excoffier & Lischer, 2010). The significance was tested using 10,000 permutations. The spatial genetic pattern was examined by spatial analysis of molecular variance (SAMOVA) using SAMOVA V1.0 (Dupanloup, Schneider, & Excoffier, 2002).

### 2.5 Demographic history analysis

To evaluate the population history of *D. punctatus*, we calculated Tajima's *D* (1989) and Fu's *F*s (1997) neutrality test statistics based on the mitochondrial DNA dataset and calculated the significance with 10,000 simulations (Rogers & Harpending, 1992; Schneider & Excoffier, 1999). Mismatch distribution analysis was also conducted to detect the population expansion of the *D. punctatus* with Arlequin V3.5 software (Excoffier & Lischer, 2010); moreover, 1,000 parametric bootstrap replicates were used to test the suitability of observed mismatch distributions to the theoretical distribution under a sudden expansion model (Rogers & Harpending, 1992). When a prominent expansion event was identified, the parameter value for the mode of the mismatch distribution (*τ*) was applied to estimate time since expansion (in generations) using the equation *t* = *τ*/2*u* (Rogers, 1995). In this study, *u* was calculated as *u* = *μ* × *k* × *g*, where *μ* represents the number of substitutions per site per year (s s^−1^ y^−1^), *k* represents the average DNA sequence length, and *g* represents the generation time in years.

Bayesian skyline plots (BSPs) implemented in BEAST v2.0 were also used to evaluate the timing of the population expansion (Bouckaert et al., 2014). For both *COI* and *ITS* fragments, the program was run with three independent runs steps (30 million simulations), and we discarded the burn‐in of the first 20% steps. The evolutionary models using AIC were selected for *COI* (GTR) and *ITS1* (TVMef+I) using jModeltest2 (Darriba et al., 2012). The proposed conventional mutation rates for insect mitochondrial *COI* gene 2.3% per million years were used to estimate coalescent time (Brower, 1994). After multiple runs, similar results and convergence to stationarity were reached with effective sample size (ESS) of at least 400. The Bayesian skyline plot (BSP) was constructed for each data set using the program TreeAnnotator included in BEAST v 2.0 (Bouckaert et al., 2014).

### 2.6 Ecological niche modeling

The ecological niche model was a useful approach to visualize the population demographic history of *D. punctatus* during the Quaternary, especially LGM. Climatic data from much earlier glaciation could not be obtained; hence, we used present and LGM climatic data. If the distribution pattern of *D. punctatus* was related to historical climatic changes, the distribution range would be expected to have obvious contracted during the LGM period (Carstens & Richards, 2007).

A total of 107 occurrence records were collected for niche modeling analysis, including 23 records from the specimens in this study and records from published documents. Bioclimatic variables were downloaded from the WordClim website (V1.4; http://www.worldclim.org/). We compared the suitable habitats and potential distributions of *D. punctatus* in two periods: the present day and the LGM. The environmental layer of the present and LGM were built at a resolution of 2.5 min, and the LGM dataset was based on the Community Climate System Model (CCSM). A distribution was generated using 15 climatic variables from the WorldClim database (all bioclimatic variables except for BIO7, 8, 17, and 18) for the current climate, with an estimate based on other distribution localities that were collected (Hijmans, Cameron, Parra, Jones, & Jarvis, 2005). The ecological niche model was constructed using the maximum entropy machine learning algorithm implemented in MAXENT (V3.3.3k; Phillips, Anderson, & Schapire, 2006), which proved to perform well compared with other methods, especially those with relatively smaller sample sizes. Analysis with default program settings (cumulative output, convergence threshold (1 of iterations (500)) was executed in ten replicates with default settings; 70% of the sites were set to train the model, and 30% of the sites were set to test the model predictions. Model performance was evaluated by both the area under the curve (AUC) of the receiver operating characteristic (ROC) plot and the binary omission rate over ten replicate runs. Area under the curve is a composite measure of model performance, which can equally weigh the omission error and commission error, and has an index of suitability between 0 and 1. The value of AUC that is >0.5 indicates the predicted results of the model are better than those predicted stochastically, and an AUC > 0.9 usually indicates excellent predictive power (Ye, Zhu, Chen, Zhang, & Bu, 2014).

### 2.7 Populations identifying with distance‐based barcoding methods

The distance between intraspecific variation (the DNA barcoding gap) is considered an important criterion in DNA barcoding. To explore the feasibility of populations that identify within *D. punctatus*, DNA barcoding gap analysis was performed based on distance‐ and character‐based methods (Dai et al., 2012). The “best close match” (BCM) (Meier, Shiyang, Vaidya, & Ng, 2006) and the minimum distance (MD) (Zhang et al., 2012) are two distance‐based methods that proved to be effective in species identification in DNA barcoding studies. To examine whether the correct local populations could be identified with these methods, we therefore performed the two protocols with “single‐sequence‐omission” or “leave‐one‐out” simulations. Here, we removed one sequence at a time and used it as a query, with all other sequences remaining as the reference database. We performed 500 random replications for each dataset. Moreover, several species of *Dendrolimus*, *D. superans*, *D. kikuchii*, and *D. houi* were chosen for interspecific level identification. We fully investigated success rates of barcoding identification at both interspecific and intraspecific levels with each single barcode, with their corresponding 95% confidence intervals (CIs) computed using equation (20) from Zhang et al. (2012). The BCM and MD were performed following the protocol of the program TaxonDNA and package MD (Meier et al., 2006; Zhang et al., 2012).

## 3 RESULTS

### 3.1 Genetic variation analyses

A total of 45 polymorphic sites were detected in the *COI* dataset, which contained 43 parsimony informative sites. These polymorphic sites defined 51 unique haplotypes (H1–H51) from the 236 sampled individuals of *D. punctatus*, 18 of which were found in single individuals. A quarter of haplotypes (15 of 51) were unique to single populations, while others were shared (Table 1). H2 was the most abundant haplotype shared by 33 individuals, mainly distributed in the southwest region of China, followed by H6, which also occurring in six populations. Overall nucleotide diversity was *π* = 0.0075 and varied across populations (ranging from 0.0011 to 0.0082). Within all populations, GZXY (see population abbreviation in Table1) had the highest *π* value (Table 1). Haplotype diversity was *h* = 0.952 (ranging from 0.000 to 0.889) at the species level, and ZJLX (see population abbreviation in Table1) had the highest *h* value (Table 1). Within‐population gene diversity (*H*_S_) was much lower than total gene diversity (*H*_T_) (0.694 and 0.942, respectively, Table 2).

**Table 2:** Estimate of nucleotide diversity (*Pi*), haplotype diversity (*Hd*), total gene diversity (*H*_T_), average gene diversity within populations (*H*_S_), interpopulation differentiation (*G*_ST_), and the number of substitution types (*N*_ST_) (mean + *SE* in parentheses) as indicators of mtDNA haplotypes and ITS ribotypes diversity

	*Pi*	*Hd*	*H*_T_	*H*_S_	*G*_ST_	*N*_ST_
Mitochondrial DNA						
*Dendrolimus punctatus*	0.00751	0.952	0.947 (±0.0204)	0.692 (±0.0424)	0.269 (±0.0383)	0.580 (±0.0486)**
South Region	0.00858	0.912	0.916 (±0.0465)	0.580 (±0.1001)	0.367 (±0.0945)	0.587 (±0.0829)*
Southeast Region	0.00643	0.942	0.960 (±0.0203)	0.743 (±0.0440)	0.226 (±0.0415)	0.527 (±0.0597)**
Internal Transcribed Spacer 1 (*ITS1*)						
*Dendrolimus punctatus*	0.00838	0.773	0.743 (±0.0649)	0.595 (±0.0596)	0.199 (±0.0635)	0.373 (±0.0783)**
South Region	0.00853	0.900	0.550 (±0.0808)	0.547 (±0.0933)	0.006 (±0.0447)	0.086 (±0.0382)**
Southeast Region	0.00688	0.630	0.916 (±0.0308)	0.735 (±0.0638)	0.197 (±0.0756)	0.457 (±0.0960)**
Internal Transcribed Spacer 2 (*ITS2*)						
*Dendrolimus punctatus*	0.00277	0.818	0.840 (±0.0350)	0.587 (±0.0496)	0.301 (±0.0633)	0.331 (±0.0763)^NS^
South Region	0.00284	0.822	0.882 (±0.0453)	0.517 (±0.0915)	0.414 (±0.1015)	0.440 (±0.1027)^NS^
Southeast Region	0.00268	0.795	0.801 (±0.0526)	0.666 (±0.0571)	0.169 (±0.0775)	0.165 (±0.0789)^NS^

Abbreviation: NS, not significant.

* *p* < 0.05, significant.

** *N*_ST_ is significantly different from *G*_ST_ (*p* < 0.01).

NS *p* > 0.05, not significant.

The length of aligned *ITS1* sequences was 670 bp including 69 polymorphic sites. Thirty‐eight different *ITS1* haplotypes (ribotypes1) were recovered (R1–R38), two of which occurred in single populations separately. Meanwhile, the other haplotypes were shared by more than two populations. The most widespread ribotypes1 was R26 occupied by 19 populations. Ribotype1 diversity was estimated to be *h* = 0.773 at the species level, ranging from 0.000 to 1.000 in different populations. At the species level, nucleotide diversity was 0.008 (*p* = 0.022), but it varied across populations, ranging from 0.000 to 0.0189, with the GDXN (see population abbreviation in Table1) population having the highest nucleotide diversity. Similar results were detected in the *ITS2* sequences dataset including 22 polymorphic sites in 181 individuals. A total of 29 different *ITS2* haplotypes (ribotypes2) were defined (N1–N29). Overall nucleotide diversity was 0.0028, and haplotype diversity was 0.818 (ranging from 0.167 to 1.000). N15 was the most popular ribotypes2, which was found in 16 populations of the southern region. Similar results were detected comparing with the mitochondrial dataset. Within‐population gene diversity (*H*_S_) was lower than total gene diversity (*H*_T_) (0.156 and 0.879, respectively; Table 2); however, only two populations harbored single ribotype for the *ITS1* dataset, and only one population for *ITS2* sequences.

### 3.2 Phylogenetic relationships and lineages divergence

**Figure 3:**
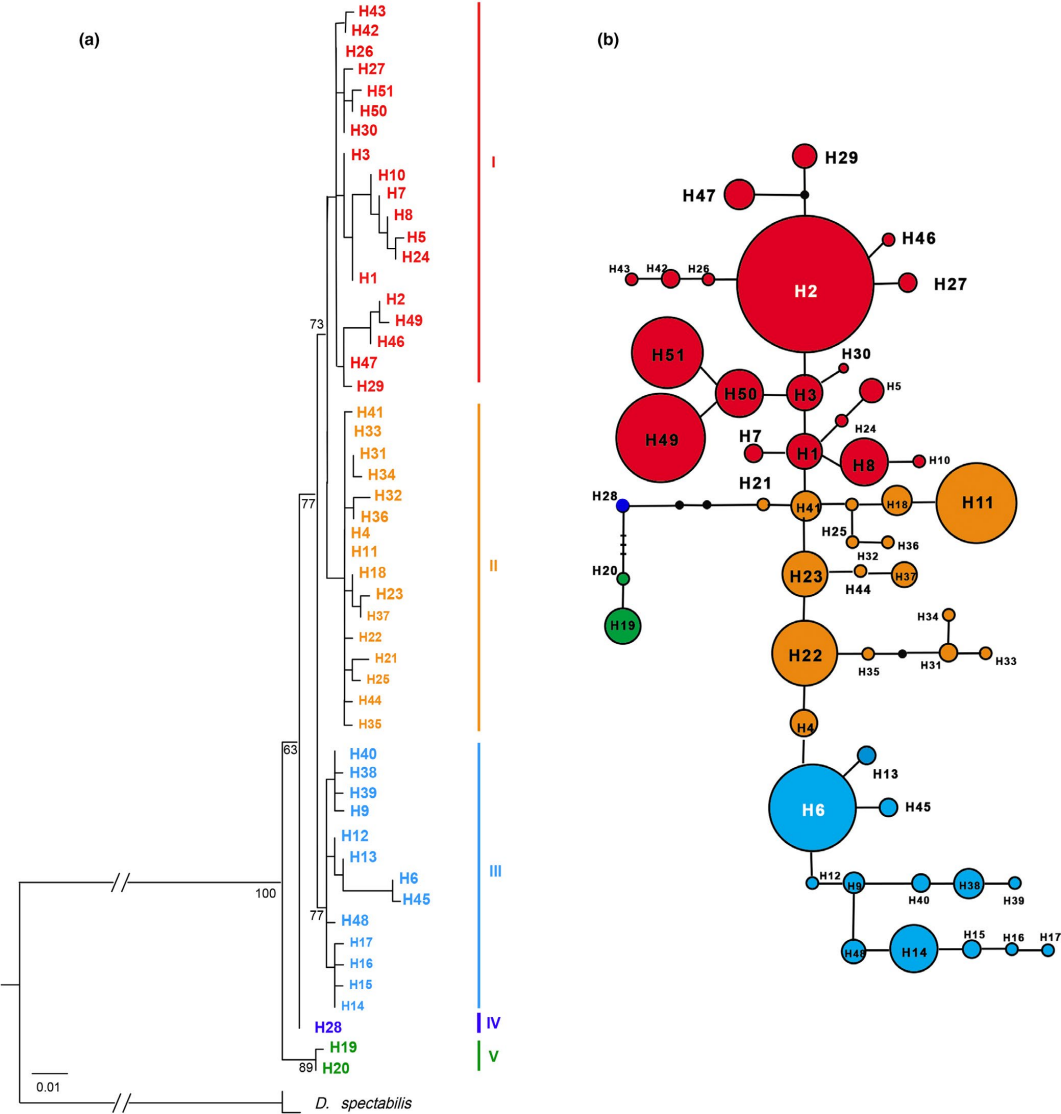
Phylogenetic relationships obtained by analysis of mtDNA haplotypes. (a) ML tree with numbers above the branches indicating bootstrap values greater than 50% for ML analysis. (b) NETWORK‐derived genealogical relationship. The sizes of the circles in the network are proportional to the observed frequencies of the haplotypes. The small black bars represent mutation steps, and the gray dots represent missing haplotype. For both subfigures, different colors represent different haplotype clusters

Phylogenetic analyses were performed using the ML and BI methods. The GTR+G model was selected by *COI* and *ITS* datasets. Based on the analysis of mitochondrial sequences, the topology of phylogenetic trees based on two methods were similar (Figure 3a). The haplotypes of *D. punctatus* were split into several discrete clades. The monophyly of *D. punctatus* was well supported. These 51 haplotypes were clustered into three multiple haplotype clades (Clades I, II, and III) and two monotypic clades (Clades IV and V) (Figure 3a). The haplotype network (Figure 3b) displayed a more detailed relationship among 51 haplotypes. The populations located in the southwest region harbored four haplotype clusters, and only two populations possessed single haplotype clusters. The haplotypes nested in Clades I and II were mostly found in the eastern part of China (Figure 2).

**Figure 4:**
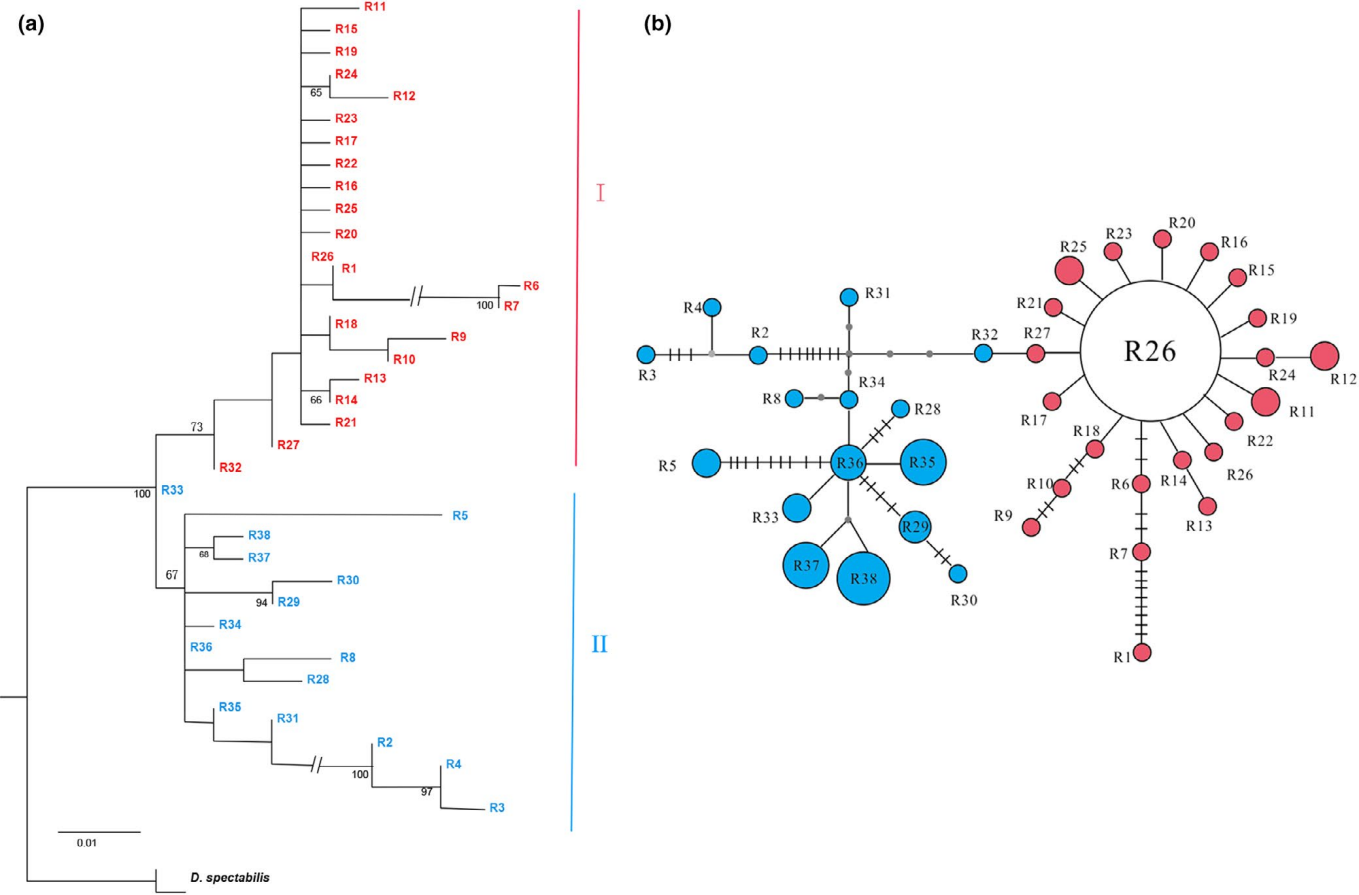
Phylogenetic relationships obtained by analysis of *ITS1* ribotypes. (a) ML tree with numbers above the branches indicating bootstrap values greater than 50%. (b) NETWORK‐derived genealogical relationship

**Figure 5:**
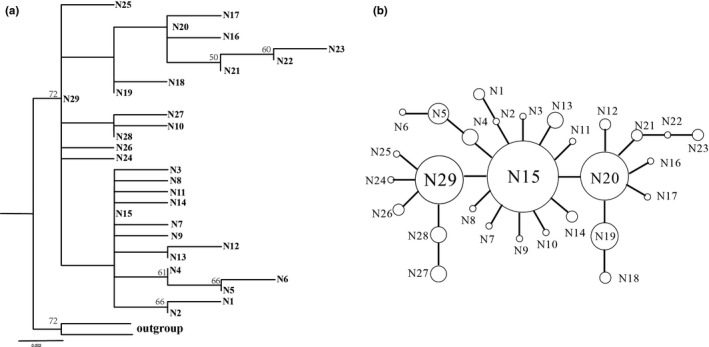
Phylogenetic relationships obtained by analysis of *ITS2* ribotypes. (a) ML tree with numbers above the branches indicating bootstrap values greater than 50%. (b) NETWORK‐derived genealogical relationship

For the *ITS1* dataset, the topological structure of the phylogenetic trees, reconstructed by two methods, were similar (Figure 4a). All 38 ribotypes were divided into two clades, in which there were 23 ribotypes1 formed a well‐support clade (Clade I). The ribotype1 network (Figure 4b) reflected similar relationships with more details, and the obvious star‐like structure network was generally interpreted as a sign that the population underwent population expansion. R26 has the highest haplotype frequency (19 of 23). Results based on *ITS2* fragment analysis agreed with *ITS1*. However, due to the relatively few polymorphic sites, the distinct topological structure was not detected based on both ML and BI phylogenetic trees (Figure 5a). Future MJ network tests showed that N15 was in the center position, which is in line with the wide distribution of N15 in 18 out of 23 populations (Figure 5b).

### 3.3 Population structure analysis

Significant genetic differentiation was identified in *D. punctatus* samples based on both mitochondrial and nuclear sequences (Table 1). Further examinations using permutation tests showed a prominent higher value of *N*_ST_ than *G*_ST_ (*p* < 0.01), indicating a strong phylogeographic structure in *D. punctatus*. The Spatial AMOVA (SAMOVA) method represents a useful method to define partitions of sampling sites that are maximally differentiated from each other without any priori assumption of population structure. However, the analysis did not clearly reveal one single group of maximally differentiated populations, as *F*_CT_ values increased progressively with increasing values of *K* (*F*_CT_ values ranged from 0.217 to 0.344 for *COI*, from 0.313 to 0.447 for *ITS1*, and from 0.193 to 0.499 for *ITS2*); however, the first level of divergence (*K* = 2) revealed a well‐defined group including those populations from the east region of China with a high frequency of mitochondrial haplotype Clade I, which were differentiated from the remaining populations. The composition of the groups detected by SAMOVA that had increasingly larger values of *K* mainly corresponded to the geographical distribution of haplotypes (Figure 2). Consistent results of two distinguished groups were confirmed by *ITS1* sequence analyses. Thus, we divided the 23 populations collected into two geographic groups: one for the eastern of China, and the other for the southwest of China. Pairwise *F*_ST_ was all significant (*p* < 0.01) in 1,000 permutation tests between two geographic regions: 0.393 for *COI*, 0.363 for *ITS1*, and 0.523 for *ITS2*, respectively. This suggested high genetic differentiation between these two geographic groups.

### 3.4 Demographic history of *D. punctatus*

**Figure 6:**
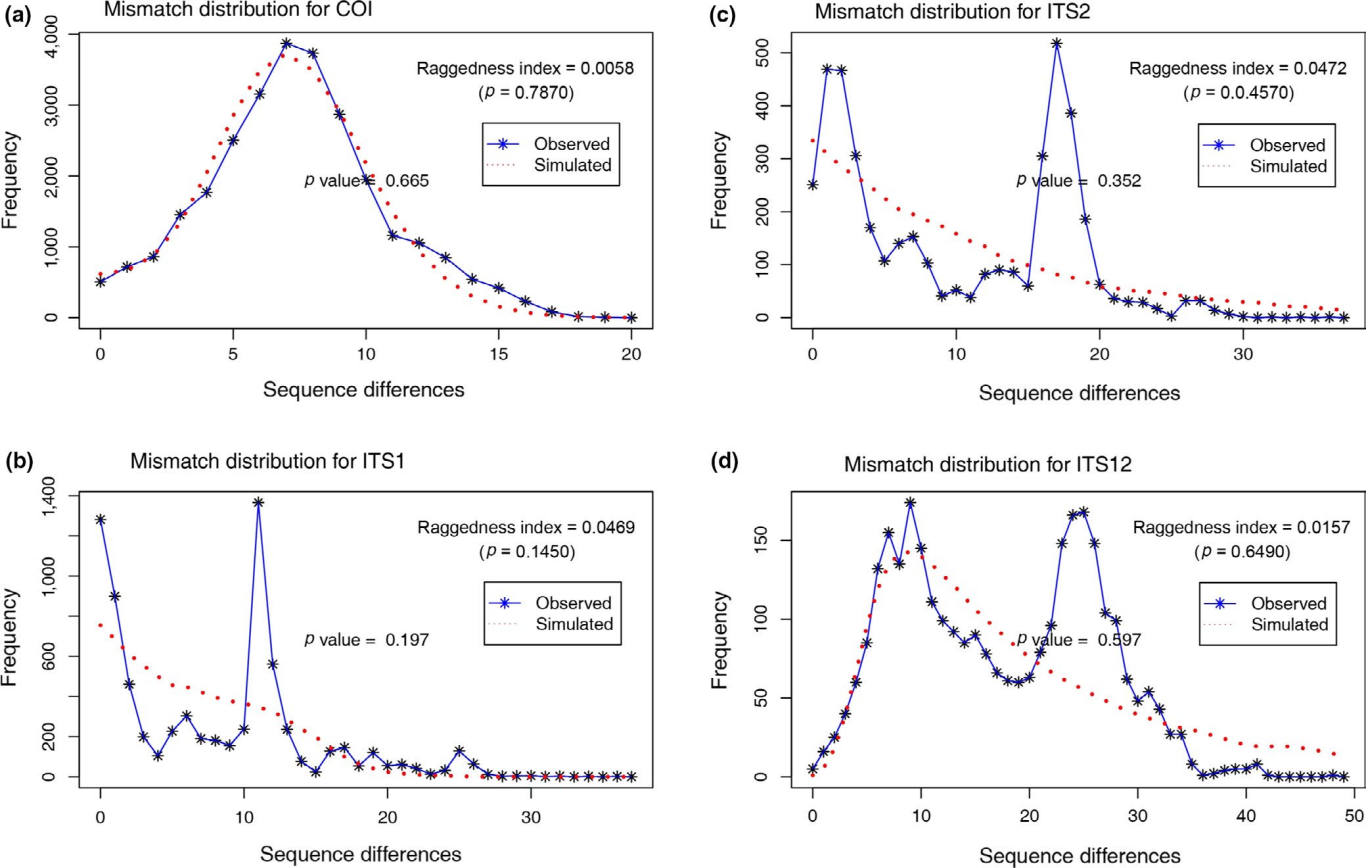
Mismatch distribution analysis based on (a) *COI*, (b) *ITS1*, (c) *ITS2*, and (d) the combination of *ITS1* and *ITS2*

**Figure 7:**
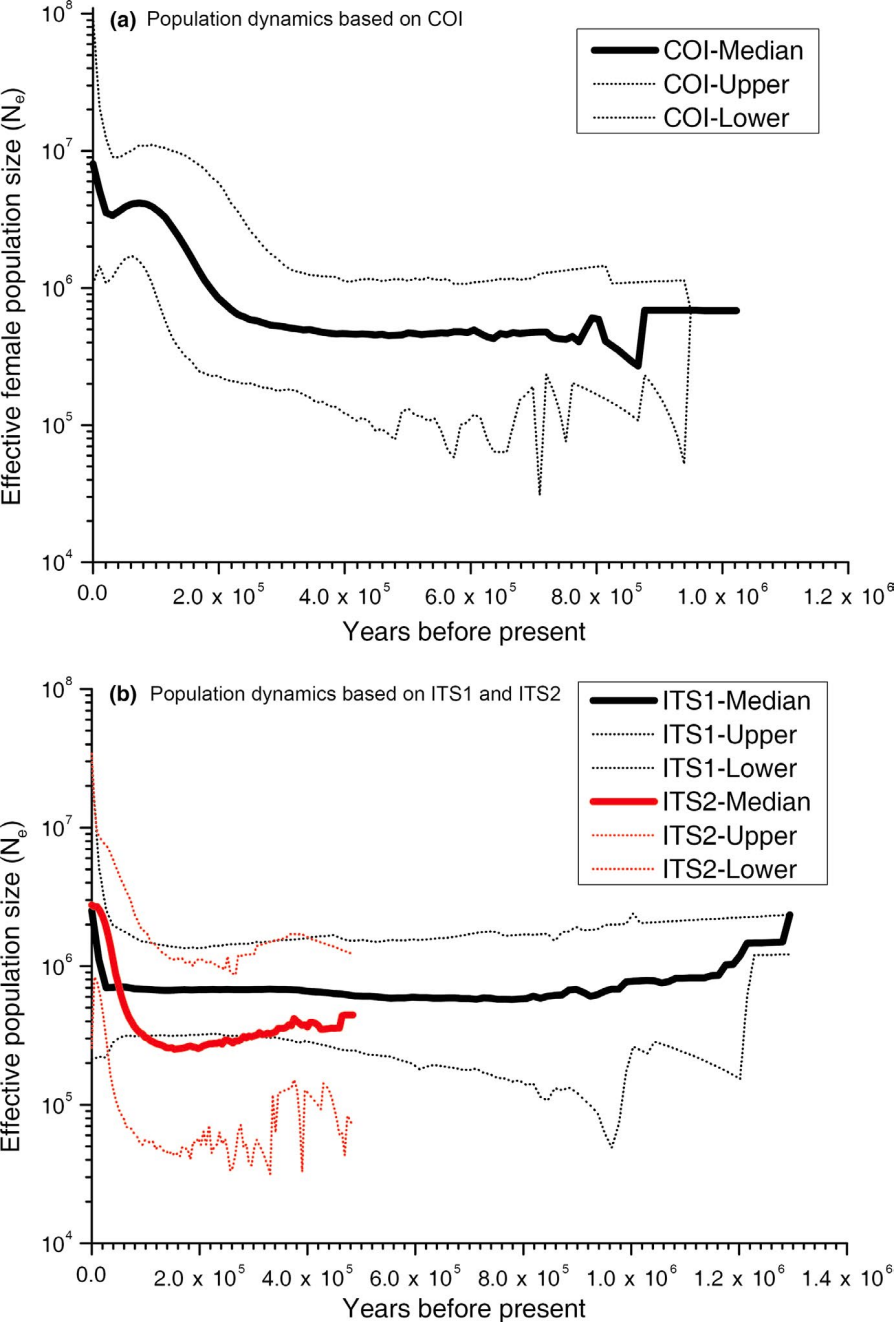
Population dynamic analysis of *Dendrolimus punctatus* Bayesian skyline plots for (a) *COI* gene and (b) *ITS* loci

As previously mentioned, a star‐like structure in a network analysis is generally interpreted as a sign of population expansion/rapid growth (Rogers & Harpending, 1992). The haplotype network results indicated historical population expansion in the masson pine moth history. This finding was further supported by the mismatch distribution analysis. Based on *COI*, *ITS1*, *ITS2*, or combined *ITS* datasets, the null hypothesis that showed sudden population expansion historically existed could not be rejected (*p* = 0.427 for *COI*; *p* = 0.049 for *ITS1*; *p* = 0.409 for *ITS2*; *p* = 0.752 for the combined *ITS1* and *ITS2*; Figure 6). However, the raggedness indices and observed variance (SSD) were not significantly different from the expectation that suggested strong and wide range gene flow occurred in the population demographic history. In addition, Tajima's *D* and Fu's *F*s were also significantly negative (Table 3), which indicated that the population of *D. punctatus* underwent rapid expansion. The estimated time of this expansion was 0.232 Mya (Table 3). The population expansion of haplotype Clade I and Clade II were also detected based on mismatch distribution analysis, as well as the Bayesian population dynamic analysis (Table 3). The results obtained from the Bayesian population dynamic analysis for all individuals are shown in Figure 7, which indicates that the population experienced conspicuous expansion, in addition to one obvious bottleneck that occurred about 0.02 Mya. For the *ITS* loci, recent population expansion was inferred at 0.025 Mya (*ITS1*) and 0.17 Mya (*ITS2*) (Figure 7).

**Table 3:** Values for *τ*, SSD, and RAG (with probability *p* values) from mismatch analysis of mitotype variation, values for Tajima's *D* and Fu's *F*_S_ (with *p* values)

Groups	Mismatch distribution						Neutrality tests	
	*τ*(*t*)	*t1–t2* (Mya)	SSD (*p* value)		RAG (*p* value)		Tajima'*D* (*p* value)	Fu's *F*_S_ (*p* value)
*Dendrolimus punctatus*	4.826	0.232–0.358		0.003 (0.493)		0.006 (0.665)	−0.180 (0.561)	−17.827 (0.001)**
Western region	2.351	–		0.014 (0.381)		0.024 (0.538)	0.686 (0.789)	−4.083 (0.100)^NS^
Eastern region	5.078	0.244–0.375		0.004 (0.346)		0.015 (0.572)	−0.476 (0.739)	−25.080 (0.000)**
Clade I	3.262	0.157–0.242		0.001 (0.907)		0.012 (0.962)	−0.281 (0.459)	−6.351 (0.024)*
Clade II	1.117	0.057–0.083		0.004 (0.537)		0.062 (0.117)	0.203 (0.108)	−6.620 (0.001)**
Clade III	1.839	–		0.006 (0.712)		0.051 (0.151)	1.123 (0.217)	−1.619 (0.076)^NS^
Clade V	0.286	–		0.0004 (0.214)		0.265 (0.345)	−1.006 (0.201)	−0.095 (0.369)^NS^

Note

Related value of Clade IV was not calculated due to insufficient individuals (2 sequences).

Abbreviations: g, estimate of population exponential growth rate; NS, not significant; RAG, the Harpending's raggedness index; SSD, sum of squared deviations; *t1*, time in million years derived from 0.0115 s s^−1^ y^−1^; *t2*, time in million years derived from 0.0177 × 10^−6^ s s^−1^ y^−1^; *τ*, time in number of generations elapsed since a sudden expansion episode.

* *p* < 0.05.

** *p* < 0.01.

NS *p* > 0.05.

### 3.5 Species distribution modeling

**Figure 8:**
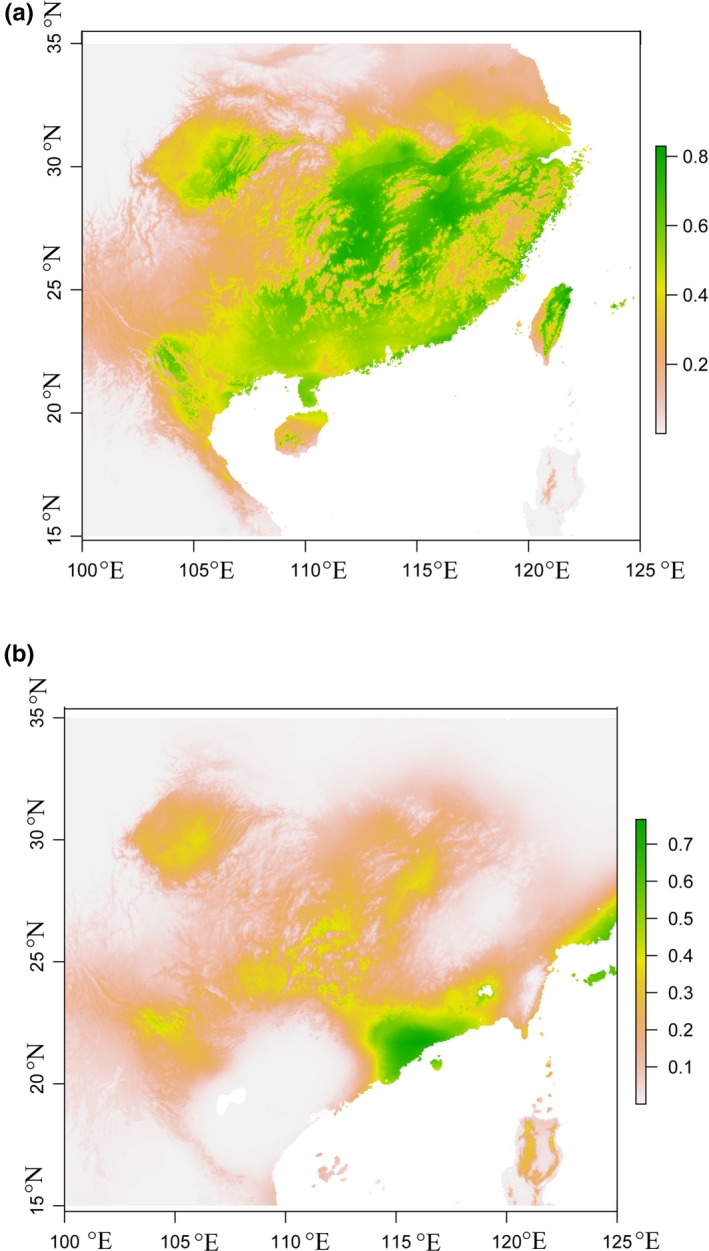
Ecological niche modeling for *Dendrolimus punctatus* during the present day (a) and the LGM (b). Different colors correspond to different fitting indices with low in red and high in green

For this study, ecological niche modeling was used to explore the relationships between genetic diversity and possible glacial refugia. A species distribution model for *D. punctatus* was built and the average AUC value was 0.917, which is considered to correspond to a useful predictive model. Under the present climatic conditions, the model revealed high climatic habitat suitability for *D. punctatus* across the south region of China. For the predicted suitable distribution, there was a significant difference between the present and LGM periods. The distribution area was contracted to the southern and southeast parts of China in LGM; therefore, the two lineages were isolated in the glacial periods (Figure 8). Interestingly, the results also revealed that the southern and southwest regions in China may have served as feasible refugia during the maximum of the Pleistocene glaciations. These findings further support the conclusions drawn by population demographic history analysis.

### 3.6 DNA barcoding in population identification

**Figure 9:**
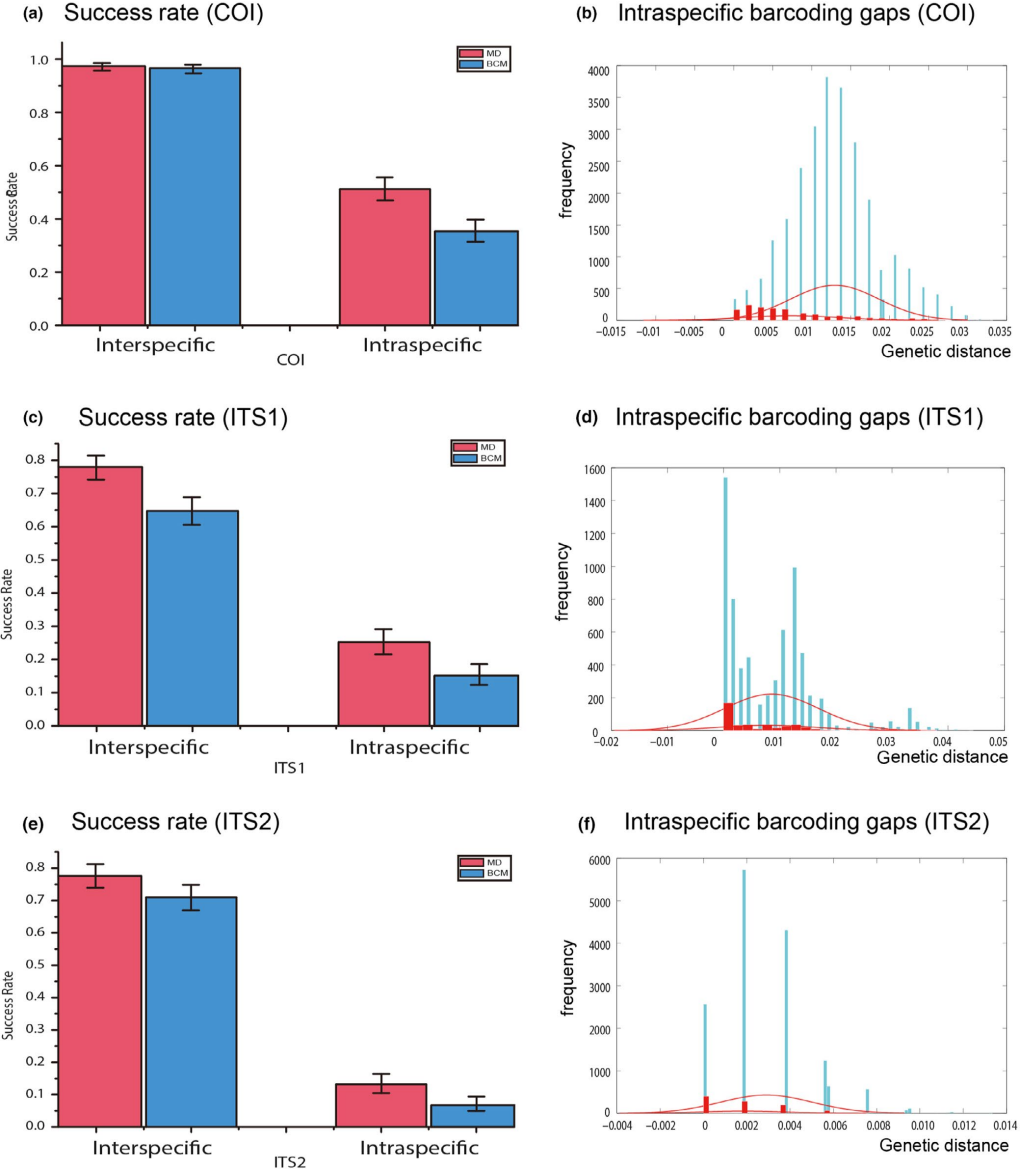
Interspecific and intraspecific DNA barcoding success rates of *Dendrolimus punctatus* and related species. (a) Intraspecific barcoding gap analysis based on *COI* gene. Blue and red histograms represent distributions of between‐ and within‐population genetic variations (K2P distance), respectively, with red fitted normal distribution curve each. (b) Interspecific and intraspecific barcoding success rates based on *COI* barcode with MD and BCM methods; (c) Intraspecific barcoding gap analysis based on *ITS1* gene; (d) Interspecific and intraspecific barcoding success rates based on *ITS1* with MD and BCM methods. (e) Intraspecific barcoding gap analysis based on *ITS2* gene; (f) Interspecific and intraspecific barcoding success rates based on *ITS2* with MD and BCM methods

In general, a large DNA barcoding gap makes species/population discrimination possible and simple. Conversely, small or negative DNA barcoding gaps blur species/population boundaries and hamper species/population assignation in DNA barcoding. The *COI* barcode showed an average between‐population K2P distance of 0.013, which was about 1.78 times larger than the mean within‐population distance. However, no positive DNA barcoding gap for the *COI* barcode (Figure 9a) was detected, indicating the difficulty of distinguishing subpopulations of this species. Both *ITS1* and *ITS2* genes presented slightly larger between‐population genetic variation (0.0085 for *ITS1*; 0.0029 for *ITS2*) compared with genetic variation within population (0.0077 for *ITS1*; 0.0018 for *ITS2*). The former was roughly 1.1 (1.1038) and 1.6 (1.6111) times larger than the latter. Similarly, there were no positive barcoding gaps or a blurred discriminations among subpopulations with *ITS1* and *ITS2* (Figure 9c and e).

Significant differences in success rates between interspecific and intraspecific barcoding identification were detected with MD and BCM methods (Figure 9b, d, and f). Intraspecific success rates were significantly smaller than interspecific ones. For instance, based on the *COI* gene, mean intraspecific success rate was 51.20% with a 95% confidence interval (CI) of 46.83%–55.55% (MD), 35.40% (95% CI: 31.33%–39.68%) (BCM), while corresponding interspecific success rates achieved values of 97.40% (95% CI: 95.60%–98.47%) and 96.60% (95% CI: 94.62%–97.87%), respectively (Figure 9b). Significantly smaller success rates were detected for nuclear markers ITS1 and ITS2, which obtained a low success rate of 25.20% (95%CI: 21.59%–29.18%) and 13.20% (95%CI: 10.51%–16.44%), respectively. The MD method (Figure 9d and f) reflected the difficulties in local population DNA barcoding.

## 4 DISCUSSION

### 4.1 Genetic diversity and population structure in *D. punctatus*

Our survey of mtDNA variation of *D. punctatus* detected several phylogeographical groups within this hazardous insect across the south region of China. This finding suggests that in the past, the distribution of this species was fragmented into several isolated refugia, where the genetic divergence of mtDNA took place. However, due to the relative limited informative sites, no significant phylogeographical pattern was revealed for *ITS2* dataset.

The mtDNA diversity was relatively high (*H*_T_ = 0.947) in *D. punctatus*, which might reflect a longer evolutionary history and the wide distribution of this species. However, the AMOVA analysis based on *COI* dataset revealed genetic differentiation among all 23 populations and most variation was from within populations (47.75%), followed by variation among groups (28.74%) (Table 4). Consequently, most of the species’ diversity was due to population divergence in which distinct phylogeographical structure signals were detected (*N*_ST_ = 0.580 > *G*_ST_ = 0.269, *p* < 0.01).

**Table 4:** Analysis of molecular variance (AMOVA) conducted on mtDNA haplotypes variation and *ITS* ribotypes for within and among populations of *Dendrophilus punctatu*

Source of variation	*df*	Mitochondrial DNA				ITS1					ITS2				
		SS	VC	PV (%)	Fixation index	*df*	SS	VC	PV (%)	Fixation index	*df*	SS	VC	PV (%)	Fixation index
Among regions	1	254.823	1.019	28.74	*F*_SC_ = 0.565**	1	136.149	1.269	30.31	*F*_SC_ = 0.282**	1	89.804	0.548	27.34	*F*_SC_ = 0.486**
Among populations	21	220.384	0.956	23.51	*F*_ST_ = 0.554**	21	126.465	1.238	25.97	*F*_ST_ = 0.363**	21	107.660	0.656	37.02	*F*_ST_ = 0.524**
Within populations	213	386.187	1.782	47.75	*F*_CT_ = 0.217*	99	201.902	1.645	43.72	*F*_CT_ = 0.313*	158	105.724	0.599	35.64	*F*_CT_ = 0.193^NS^
Total	235	861.394	3.757			121	464.516	4.152			180	303.188	1.803		

Abbreviations: *df*, degrees of freedom; *F*_CT_, correlation within groups relative to total.; *F*_SC_, correlation within populations relative to group; *F*_ST_, correlation within populations relative to total; NS, not significant; PV, percentage of variation; SS, sum of squares; VC, variance components.

* *p* < 0.05, significant.

** *N*_ST_ is significantly different from *G*_ST_ (*p* < 0.01).

NS *p* > 0.05, not significant.

Further tests carried out with *ITS1* were corroborated with our *COI* findings, and distinct phylogeographic signals (*N*_ST_ > *G*_ST_, *p* < 0.01) were found in entire distribution regions, including western and eastern regions. A relatively low value of total diversity was recorded for *ITS* dataset in *D. punctatus* (*ITS1*: *H*_T_ = 0.743, *ITS2*: *H*_T_ = 0.840), which was lower than the mitochondrial dataset, largely due to reduced genetic divergence (*ITS1*: *G*_ST_ = 0.199, *N*_ST_ = 0.373; *ITS2*: *G*_ST_ = 0.331, *N*_ST_ = 0.301), as within‐population diversity was also decreased compared with mtDNA (*H*_S_ = 0.595 for *ITS1* and *H*_S_ = 0.587 for *ITS2*). The primary cause of this discrepancy is due to the different modes of inheritance, maternal for mtDNA and biparental for *ITS*. The latter method leads to higher levels of gene flow. Compared with the protein‐coding *COI* region, the noncoding *ITS* marker was more sensitive at detecting gene flow among interbreeding populations. Similar inconsistencies were shared in many other Lepidopteran insets, for example, *Biston suppressaria* (Cheng, Jiang, Xue, et al., 2016; Cheng, Jiang, Yang, et al. 2016), *Apocheima cinerarius* (Liu et al., 2016), *Biston panterinaria* (Cheng, Jiang, Xue, et al., 2016; Cheng, Jiang, Yang, et al., 2016), and *Hyles euphorbiae* (Mende & Hundsdoerfer, 2013).

The AMOVA analysis of the mtDNA data revealed that within‐population variation was greater than among‐region variation. Nevertheless, *D. punctatus* is split into two major genetic lineages based on the analysis of mtDNA and *ITS1* (*F*_CT_ = 0.127 and 0.313, separately) (Table 4). This phenomenon could be explained as repeated population expansion and contraction caused by climate oscillations during the ice age, which resulted in lineage differentiation and limited gene flow. Physical barriers and ecological climate factors usually played a key role in obstructing gene flow among populations (Fjeldså, Bowie, & Rahbek, 2012). Although subtropical China has never been covered by extensive ice sheets during the ice age, iterative alternation of glacial and interglacial periods particularly caused distribution changes of both plants and animals (Liu, 1988; Qiu et al., 2011). Continuous and intensive tectonic activities had already begun in subtropical China during the Miocene and Pliocene (Webb & Bartlein, 1992), which made a great effort in shaping the distribution and differentiation of local organism. The northeast–southwest trending mountain ridges in subtropical China, such as Mt Wu, Mt Xuefeng, and Mt Wuyi, acted as barriers against the inflow of cold air from the northwest and maintained warm moist air coming from the Pacific, resulting in a humid climate in the eastern region and relative arid environment in the southwest. Moreover, the Qinghai‐Tibetan Plateau (QTP) and Mt Hengduan obstructed the dampness from the Indian Ocean, which further exacerbated the drought in this region. Mt Xuefeng also acted as a protective barrier against different climate environments on the eastern edge of the Yungui Plateau (YGP) (Holt et al., 2013). Restricted gene flow between the northwestern and eastern lineages was likely to be affected by these ridges. These mountains were important faunal dividing criterions in subtropical China. Similar geographical separations have been reported in many pervious researches, including the *Leucodioptron canorum* (Li et al., 2009), *Squalidus argentatus* (Yang et al., 2012), and *Microvelia horvathi* (Ye et al., 2014).

Meanwhile, present limited gene flow between southwest and east regions was also caused by the decreased ability of long‐distance dispersal by *D. punctatus* (Hou, 1987). As a typical phytophagous insect with intermediate mobility, *D. punctatus* exhibited a steep genetic decline with distance. The populations around the distributional center tended to have higher levels of genetic variations than the peripheral ones (Table 1; Figure 2). High genetic diversity in the center of the range of distribution reflected that the gene flow was strong among closely situated populations, which apparently was due to its ability to disperse in nearby areas. Geographic barriers such as Mt Wu, Mt Xuefeng, and Yungui Plateau (YGP) weakened the population diffusion and gene flow of *D. punctatus*, especially between the southwest and east regions.

### 4.2 Pleistocene range expansion and potential refugia of *D. punctatus*

High polymorphism detected in the *D. punctatus* with mtDNA and *ITS* may reflect the wide distribution of this forest pest in southern China, with large and relatively stable population sizes. This was compared with other pine pests distributed in Europe (e.g., *Dendrolimus sibiricus*, *Dendrolimus pini*, and *Dendrolimus sibiricus*), which experienced drastic reduction in population size due to the coverage of ice sheet during the Quaternary (Kononov et al., 2016; Mikkola & Ståhls, 2008).

Both our phylogenetic and network analyses showed that *D. punctatus* have experienced population expansion in subtropical China. According to mismatch distribution and Bayesian population dynamic analysis based on the mtDNA dataset, the population expansion started at ca. 0.23 Mya. Cui et al. (2011) reported a detailed partition criterion of Quaternary glaciations in China and concluded that six major glaciations occurred since Wangkun Glaciation (0.7–0.5 Mya). The expansion of *D. punctatus* initiated at the interglacial period of Guxiang Glaciation (Penultimate Glaciation) (also known as MIS7). During the interglacial periods of Penultimate Glaciation, populations could be effectively preserved in the mountainous areas of southern China. In addition, *D. punctatus* is one of the most serious and economically damaging insect pests, which have the genetic characteristics of population outbreak initiated by high temperature and drought (Zhang et al., 2004). The interglacial periods provide feasible climate conditions for the population outbreak and allow the maintenance of relatively large population sizes, thus retaining high genetic diversity. Interestingly, the event of demographic bottlenecks was detected based on the Bayesian skyline plot analysis of mtDNA dataset. This sudden population shrink occurred at ca. 0.02 Mya, which belong to the LGM period.

*Dendrolimus punctatus* is a typical phytophagous insect, in which its patterns of populational distribution and dynamics have significant correlation not only with the meteorological factors but also its host plants. Although the main host plant of *D. punctatus* is the masson pine, *P. massoniana* and its caterpillars have been reported to feed on other conifers, such as *Pinus thunbergii*, *Pinus kwangtungensis*, and *Pinus taeda* (Cai, 1995; Hou, 1987). The extensive hilly areas of subtropical China (especially between 22°N and 34°N) provide advantages to harbor temperate deciduous forests and coniferous (Yu et al., 2000). Many researchers have argued that lots of local plant species experienced long‐term fragmentation and refugial survival, such as *Cathaya argyrophylla* (Pinaceae) and *Eurycorymbus cavaleriei* (Sapindaceae), and several postulating separate refugia (such as Mts Dayao, East Yungui Plateau, and Mts Nanling) in subtropical China (Qiu et al., 2011; Wang, Gao, Kang, Lowe, & Huang, 2009). In addition, clear phylogeographic histories of *P. kwangtungensis* (Pinaceae), a closely related species of the host plant *P. massoniana*, were demonstrated among group *F*_ST_ differentiation, suggesting that Yungui Plateau and the Mts Nanling were a major refugium (Tian, López‐Pujol, Wang, Ge, & Zhang, 2010). Furthermore, more recent studies have confirmed that *P. massoniana* also existed in multiple glacial refugia fostering populations with high genetic diversity (Ge et al., 2012; Zhou et al., 2010). The high genetic variation of *D. punctatus* in the southwest region is an alternative explanation for the retention of ancestral polymorphisms. A similar genetic divergence pattern was also detected in our study. The populations form Yungui plateau (GZXY and GZHZ, see population abbreviation in Table1) and Mts Guangzhou Province (FJSH, see population abbreviation in Table1) harbored haplotypes from separate haplotype clusters, suggesting a shared semblable refugia with its host plants.

Temperate evergreen forest, such as *P. massoniana*, recolonized the hilly regions of south China because of the increasing temperature since the Holocene (Harrison et al., 2001). Increased number of host plants provided great opportunity for sustaining population expansion of *D. punctatus*. In our study, a noteworthy trend revealed higher diversity in the southern region than in the east and north regions, reflecting a common scenario of postglacial, northward recolonization. Two stages of population growth are also well consistent with the last interglacial high sea level (MIS5, 0.13–0.07 Mya) and postglacial‐Holocene warm climate (0.018–0 Mya). The warm climate and high sea level influenced interglacial continental vegetation distribution.

The extinction of the populations in the northern parts of China, caused by migration to the south during the glacial maximum, might have survived in separate refugia. Our survey of mtDNA variation throughout the geographical distribution of *D. punctatus* suggests that several isolated refugia were existed where mtDNA divergence occurred. In addition, population dynamic analysis indicated that the *D. punctatus* species experienced population expansion, and an obvious northward recolonization was detected. The haplotype distribution of the present investigation seems further to indicate that the common fixture of the same haplotype among the disjunct populations was mainly caused by the founder effect during the population recolonization in this region. The highest latitude population (pop 23, HBCD, see population abbreviation in Table1) has relatively low genetic divergence even though much more individuals were used, which indicates the founder effect may be an important factor that influences genetic differentiation of *D. punctatus*.

### 4.3 Applicability of DNA barcoding at population level

To assign unknown individuals to certain populations within a species or to identify population from DNA sequences is not a new approach (Lou & Golding, 2010). Here, we present a study in which we systematically investigated the population structure and dynamic of *D. punctatus* with a variety of DNA barcoding methods, based on both commonly used *COI* gene, and two alternatives genes, *ITS1* and *ITS2*. The intraspecific success rate of barcoding identification was found to be significantly lower than the interspecific ones, indicating the difficulty in barcoding populations (Figure 9). For the *COI* gene, among 23 populations of *D. punctatus*, only one single local population (pop23, HBCD) formed a monophyletic clade (Figure 3). Moreover, compared with the *COI* gene, less population structure was also found for *ITS* genes on the phylogenetic trees (Figure 4, 5). The results may not be surprising since the coalescence time is generally shorter than the time of different species to their most common ancestor (TMCA), therefore, no large enough variation or mutations accumulated during this relatively short time period. Furthermore, The AMOVA analysis of the mtDNA data suggested that strong gene flow existed in the *D. punctatus* populations, which also impaired the success rate of population assignments and resulted in no positive barcoding gaps being detected (Figure 9).

Despite the fact that the sequence information collected from DNA barcoding was not sufficient to solve population‐level questions, it reflected genetic diversity among different population (Bazin, Glémin, & Galtier, 2006; Moritz & Cicero, 2004). It should be noted, however, that DNA barcoding markers have the valuable potential to investigate the historical population dynamics of *D. punctatus*. It would be interesting to investigate further the applicability of barcoding information in the study of genetic diversity of other species.

## 5 CONCLUSION

We investigated the population structure and dynamics of *D. punctatus* based on the genetic variation of mtDNA as well as nuclear markers. A history of postglacial recolonization of the populations and multiple refugia during the ice age of the Quaternary was inferred. Many previous phylogeographic studies have paid close attention to the effects of very large mountainous systems such as the Mts Hengduan and Mts Qinling, but less attention was paid to the effect of the smaller mountain regions in the subtropical regions of China. The findings of this study proved that several potential refugia and geographic barriers existed in these hilly areas. These results provide a phylogeographical hypothesis for further testing; however, given the small sample size and limited molecular markers, many low‐copy nuclear genes and mitochondrial genomes are required to further examine the genomic divergence within *D. punctatus*.

## CONFLICT OF INTEREST

None declared.

## AUTHOR CONTRIBUTIONS

Jing Li wrote the MS and Qian Jin performed the experiment. Jing Li and Qian Jin analyzed all the data together. Geng‐ping Zhu and Chong Jiang provided great help in ecological niche modeling analysis. Ai‐bing Zhang defined the project as the main supervisor and considered the whole analysis.

## Data Availability

DNA sequences: GenBank accession numbers: KF366914–KF367149 for *COI*, KF367150–KF367271 for *ITS1*, and KF367272–KF367452 for *ITS2*. BOLD system accession numbers: GBGL14669–GBGL14904 for *COI*.
